# Onychomycosis in Psoriatic Patients with Nail Disorders: Aetiological Agents and Immunosuppressive Therapy

**DOI:** 10.1155/2020/7209518

**Published:** 2020-05-02

**Authors:** Núbia Carvalho Pena de Oliveira Praeiro Alves, Tomaz de Aquino Moreira, Lucivânia Duarte Silva Malvino, José Joaquim Rodrigues, Roberto Ranza, Lúcio Borges de Araújo, Reginaldo dos Santos Pedroso

**Affiliations:** ^1^Postgraduate Program in Health Sciences, School of Medicine, Federal University of Uberlândia, Pará Av. 1720, Umuarama, Uberlândia, Minas Gerais, Brazil; ^2^Rheumatology Service of the Clinical Hospital of Uberlândia, Federal University of Uberlândia, Pará Av. 1720, Umuarama, Uberlândia, Minas Gerais, Brazil; ^3^Clinical Analysis Laboratory of the Clinical Hospital of Uberlândia, Federal University of Uberlândia, Pará Av. 1720, Umuarama, Uberlândia, Minas Gerais, Brazil; ^4^Federal University of Uberlândia, João Naves de Ávila Av. 120, 1F Block, Santa Monica, Uberlândia, Minas Gerais, Brazil; ^5^Technical Course of Clinical Analysis, Technical School of Health, Federal University of Uberlândia, Amazonas Av., 6X Block, Umuarama, Uberlândia, Minas Gerais, Brazil

## Abstract

Psoriasis and psoriatic arthritis are chronic, relapsing, immune-based diseases. Psoriatic patients may have nail involvement in 50 to 80% of cases, and this may reach 85% in patients with joint disease, in spite of the fact that the relationship between psoriasis and onychomycosis is not well established. The aim of this study was to investigate the occurrence of onychomycosis in patients with nail disorders and diagnosis of psoriasis and psoriatic arthritis. This was a cross-sectional study in which 38 patients diagnosed with psoriasis and/or psoriatic arthritis were interviewed and had altered nail samples analysed by mycological and histopathological exams. Twenty-two (57.89%) patients had a confirmed diagnosis for onychomycosis. Seventeen (44.8%) had a positive direct mycological examination, 16 (42.1%) had positive cultures, and 12 (31.6%) were positive for fungi by histopathological examination. Dermatophytes were identified in nine (56.3%) cultures, and of these, eight were *Trichophyton rubrum* and one *T. tonsurans*. Yeasts were isolated in seven patients (43.75%), which included four *Candida parapsilosis* and three *C. albicans*. Six patients (15.78%) were not using immunosuppressive therapy, and the others were using methotrexate, etanercept, adalimumab, infliximab, secukinumab, or golimumab, in monotherapy or in combination with other drugs. The confirmed onychomycosis rate in patients using methotrexate alone was 92.8% (*n* = 13). We concluded that it is possible that there is a positive relationship between psoriatic disease and onychomycosis. And we highlight that it is also worth investigating in the future the possible role of immunosuppressive therapy (mainly methotrexate) as a predisposing factor for the development of fungal infections in psoriatic patients.

## 1. Introduction

Onychomycosis is a nail infection caused by dermatophyte fungi, nondermatophyte filamentous fungi, and yeasts [1, 2]. It is the most common nail disease in clinical practice, causing symptoms such as local pain, paraesthesia, difficulties in daily activities, and social interactions [1]. Major clinical signs include depigmentation, hyperkeratosis, and onycholysis [3].

The worldwide prevalence of onychomycosis is 5.5%, and this increases with age [1, 4]. In a retrospective study of 8331 patients, the prevalence reached 20.7% in patients over 60 years [5]. Approximately 60–70% of infections are caused by dermatophyte fungi, most commonly *Trichophyton rubrum* (>50%) and *T. mentagrophytes* (20%), and another 30–40% have nondermatophyte filamentous fungi and yeasts as aetiological agents [1].

A natural barrier in the nails usually prevents the development of fungal infections. However, this barrier can be affected by aging, repeated nail trauma, peripheral artery disease, and various other diseases. Psoriasis is one of the diseases that causes nail abnormalities and is often difficult to differentiate from onychomycosis, in which cases a differential diagnosis must be made [6, 7].

Psoriasis is a chronic inflammatory autoimmune disease that manifests with red patches of skin covered with thick, silvery scales and has a prevalence of 2-3% in the population [6]. Psoriasis is a multiorgan disorder that usually appears in patients with associated comorbidities, such as systemic arterial hypertension and cardiovascular diseases, obesity, type 2 diabetes, nonalcoholic fatty liver disease, and anxiety [8, 9]. This disease has an impact on the patient's quality of life and affects the social relationships and psychological health and makes work-related activities difficult. The quality of life will be affected especially when the manifestations occur in visible areas of the body such as face, arms, hands, and nails, in addition to the consequent physical limitations and pain that result from the disease [8, 9].

About 50 to 80% of psoriatic patients may have nail involvement, and this may reach 85% in those with joint disease. The degree of nail involvement depends on the location of the inflammatory process, the intensity, and the time of evolution and may manifest mainly with pitting, hyperkeratosis, and onycholysis [6, 10].

The relationship between psoriasis and onychomycosis is controversial in the literature and varies according to the geographic region of the study. The frequency of onychomycosis in this patient group can range from about 4% to over 60% depending on the study [6]. The most common pathogens found in these patients also have variable frequency with some studies showing a higher occurrence of dermatophytes and others highlighting nondermatophyte filamentous fungi and yeasts [6, 7].

This study was designed to verify the presence of onychomycosis in patients with psoriatic disease treated at a public reference service in Brazil and identify the main infection agents present. We also aimed to verify the occurrence of fungal infection in patients using immunosuppressive therapy, in order to contribute to the appropriate and directed management of the disease and make it possible to contribute to the better quality of life of the patient, resulting in improved self-esteem.

## 2. Materials and Methods

This is a cross-sectional study conducted at the Psoriasis and Psoriatic Arthritis Outpatient Clinics of a hospital in Brazil. In general, this regional reference hospital is the largest provider of services by the Unified Health System (SUS) of the state of Minas Gerais and third in the ranking of the largest university hospitals of the Ministry of Education, Brazil.

Patients with previous diagnosis of psoriasis and/or psoriatic arthritis who attended for hospital care since June 2017 to February 2018 (during nine months), aged 18 years or older, and who presented nail changes clinically compatible with onychomycosis (onycholysis and/or subungual hyperkeratosis) were included in the study. All patients were evaluated by dermatologists at the outpatient clinic for selection and inclusion in the study. The patients with nail alterations not compatible with onychomycosis (pitting and pterygium), patients taking antifungal drugs, or having discontinued systemic and topical antifungals in the previous 30 days were excluded from the study.

The authors confirm that the ethical policies of the journal, as noted on the journal's author guidelines page, have been adhered to and the appropriate ethical review committee approval has been received. The study was approved by the Research Ethics Committee of the Federal University of Uberlândia (protocol number: 2.088.875/2017). All participants signed the informed consent form.

An evaluation form was applied that included questions about general information, evolution of psoriatic disease, medicines in use (including immunosuppressive therapy), previous reports of fungal infections, description of nail changes, and the number of nails that were affected. Then, the samples were collected for mycological examination after antiseptic cleaning of the nail with 70% alcohol and boric water, in an attempt to reduce the presence of contaminants. Next, nail clippings were collected for direct mycological examination, fungal culture, and histopathological analysis. This technique was chosen because it presents a minimal risk of nail dystrophy and is atraumatic, fast, and easy to perform [11, 12].

Direct mycological analysis was performed in 30% NaOH and cultures on Sabouraud-chloramphenicol agar and Mycosel® agar. Histopathological evaluation was also performed on hematoxylin-eosin and periodic acid Schiff (PAS) [2]. In the PAS, the presence of uniform septate hyphae invading the nail plate suggests dermatophyte infection, thicker tortuous wall hyphae represent nondermatophyte fungi, and conidia on the ventral surface of the lamina, especially if accompanied by buds and pseudohyphae, suggest *Candida* infection [13]; however, identification of the species causing the infection is not possible [14].

The isolates were identified by standard mycological procedures. The filamentous fungi cultures were incubated at 30°C for 10–14 days and identified using macroscopic and microscopic examination after microculture in potato agar. Yeasts were identified by colony color on chromogenic Candida agar, morphology on cornmeal Tween 80 agar, and assimilation profile done by Vitek-2 YST (bioMérieux, Marcy I'Etoile, France).

Onychomycosis was diagnosed when both direct analysis and culture were positive, except when the isolated fungus was a dermatophyte, in which cases the positive culture alone was considered sufficient for diagnosis. However, when both tests were negative, but the pathological analysis was positive, diagnoses of onychomycosis were still given. All three analyses were performed simultaneously to increase the rates of onychomycosis diagnosis.

Statistical analysis was performed by the Shapiro–Wilk normality test. Quantitative variables were described within each group as mean, median, maximum, and minimum standard deviation. Qualitative variables were described by frequency and percentage. Associations of qualitative variables were assessed by the likelihood ratio test [15]. Quantitative variables, such as age, which presented a normal distribution, were evaluated by a Student's *t*-test for association with direct mycological analysis, culture, and pathology [16]. All tests were applied using a significance level of 5% (*p* < 0.05). The procedures were performed using SPSS v.20 software.

## 3. Results

Forty-six patients had nail changes, but of these, three were excluded because they did not fit the inclusion criteria. Five patients did not undergo mycological examinations, leaving 38 patients.  Table 1 shows the characteristics of the individuals included in the study.

The age of the patients ranged from 31 to 79 years, with a mean of 53.8 years, including 20 (52.6%) females and 18 (47.4%) males. The time of diagnosis of psoriatic disease ranged from 1 to 30 years previously among patients, where 26 (68.4%) had a diagnosis for more than 10 years. Nineteen patients (50%) reported nail changes over 10 years ago.

Regarding the manifestation, 19 patients (50%) presented with both psoriatic arthritis and psoriasis, 15 (39.5%) only had psoriasis, and four (10.5%) only had psoriatic arthritis. There was a statistically significant difference between the manifestation of psoriatic disease (psoriasis or psoriatic arthritis) and onychomycosis (*p* < 0.05), but not in patients who had both forms of the psoriatic disease simultaneously, and onychomycosis. At clinical examination of the nails, 21 patients (55.3%) were identified to have changes in their toenails, six (15.8%) had changes in their hands, and 11 (28.9%) in both. The most frequent nail alteration was subungual hyperkeratosis in 13 patients (34.2%), followed by onycholysis in 11 (28.9%) and both in 11 (28.9%).

The results of mycological analyses are presented in Tables 2 and 3. Among the 38 patients, the rate of onychomycosis was 57.9% (22 patients). The most frequent fungi were from the dermatophyte group (nine patients, 56.25%), most of them (seven patients) in toenails, and two simultaneously on fingernails and toenails. Eight (50.0%) were *T. rubrum* and one *T. tonsurans*. Yeasts were identified in seven cases (43.7%), and all occurred on the fingernails but three simultaneously on fingernails and toenails. *C. parapsilosis* was the most frequent species (four patients, 25.0%). There was no growth of nondermatophyte filamentous fungi in culture.

The pathological analysis showed 12 (31.6%) positive samples for fungi, and three (7.9%) showed typical histological alterations of psoriasis. The diagnosis of onychomycosis was made exclusively by the anatomopathology analysis in six cases (27.27%), but this does not allow the identification of the fungal species present.

Regarding the treatment received for psoriatic disease, only three (7.9%) were not using systemic treatment. Most patients received methotrexate alone, *n* = 14 (36.8%). The remaining patients were using acitretin or other immunobiological agents that are associated, or not, with methotrexate (Table 4).

The use of systemic treatment and onychomycosis was positively related (*p* < 0.05). Patients without treatment or on acitretin alone (immunomodulator) did not have an increased likelihood of onychomycosis. The patients on methotrexate alone had a 92.8% positivity rate for onychomycosis (*n* = 13; *p* < 0.05). Increased rates of onychomycosis were also identified in those using adalimumab and infliximab alone. When the use of these drugs was associated with methotrexate, no increase in onychomycosis rates was observed. There was also no increase in onychomycosis positivity related to the use of other immunobiological drugs. Data are shown in Table 4.

## 4. Discussion

The coexistence of psoriasis and onychomycosis has been reported in the literature, with a suggestion that onychomycosis has a higher prevalence in psoriatic patients, 18% versus 9% in a control group, according to a systematic review by Klaassen et al. [17]. In the present study, it was observed that the frequency of onychomycosis among patients treated at the dermatology and rheumatology services of the hospital was 57%. In a multicentre study, it was found that patients with psoriasis had a higher risk for onychomycosis, 56%, when compared to the group without psoriasis, considering the same age and gender [18]. Other studies have found a prevalence of onychomycosis in psoriatic nails of 62–70% in Pleven and 52% in Plovdiv, both in Bulgaria, 43% in Thessaloniki (Greece) and 34.78% also in Thessaloniki, and 34% versus 4% in Pakistan [6, 7, 19]. As noted, there is a wide variation in frequency depending on the study and geographic region.

The nail manifestation of psoriasis causes abnormalities in the blood capillaries and leads to the loss of natural defenses against microorganisms, a fact that could facilitate infection [18, 20, 21]. In addition, nail detachment that occurs in psoriasis induces a humid environment that may facilitate fungal proliferation [22].

However, it has also been suggested that the increased turnover caused by psoriasis, with rapid growth and peeling nails, could be a factor that would prevent fungal proliferation [18]. In agreement with this, there is an increased presence of serum-like glycoproteins and inhibitor peptides that would hinder the development of microorganisms in these patients [22]. However, despite the heterogeneity of the studies, sometimes with controversial results, there is a strong indication that there may be a relationship between psoriasis and onychomycosis, as well as the frequency difference that has been observed between geographic regions [7, 17].

Treatment against psoriasis, which involves the use of immunosuppressive drugs such as methotrexate and cyclosporine, may alter the immune status of these patients, predisposing them to nail infections [18, 22]. In the present study, most patients were using immunosuppressive therapy and there was statistical significance (with *p* < 0.05) when analysing the use of immunosuppressive drugs versus the presence of onychomycosis. By analysing the use of methotrexate alone, of the 14 patients, 13 confirmed the diagnosis for onychomycosis. There was also a higher frequency of onychomycosis in patients taking adalimumab and infliximab, despite the small number of patients.

*Trichophyton rubrum*, from the dermatophyte group, was the species isolated in half of the cases. This species is generally responsible for the majority of onychomycosis cases, according to different studies. For example, Zisova et al. [7], in Bulgaria and Greece, showed positivity for dermatophyte fungi in 67% of the patients, and of these, 83% were *T rubrum*, 16% *T. mentagrophytes*, and 24% *Candida* spp. [7] However, the occurrence of yeast in nails (43.75%) in the present study was also high, especially when compared with other studies. For example, Tsentemeidou et al. [6] found yeast as an onychomycosis agent in 37.5% of patients, nondermatophytes in 37.5%, and *T. rubrum* only in 12.5% [6]. In the past, yeasts were considered contaminants and colonisers, but nowadays they are increasingly being recognised as pathogens in nail infections, in addition to some nondermatophyte fungi [19].

*T. tonsurans* is a rare agent of nail infection [23] and was isolated from the nail of a 63-year-old hypertensive diabetic patient with no history of nail trauma. In the study by Eba et al. [24], this species was the third most common isolated nail from diabetic patients.

A European project on foot diseases, called the Achilles Project, showed that the most common pathogens causing onychomycosis were dermatophytes, followed by *Candida* and *Aspergillus*, respectively [25]. Some studies have shown that the nails of psoriasis patients are more easily colonised by yeast as altered subungual tissue and onycholysis could facilitate yeast invasion [26–29].

In the present study, there was no isolation of nondermatophyte filamentous fungi. The role of immunosuppressive therapy in the growth and viability of different fungal groups is unknown, and this may lead to difficulty in comparing the results of different studies, as some studies did not include patients using this group of drugs [6]. Therefore, different hypotheses may be formulated to explain these findings, such as the possible role of immunosuppressive drugs as facilitators for the proliferation of dermatophytes and yeasts, but inhibiting nondermatophytes. Another aspect is the possibility that the use of nail clippings collects samples only from the distal portion of the nail, reducing positivity rates due to the absence of live fungal structures in the distal portion. Increased positivity could be achieved by collecting material from the proximal portion of the nails [19].

Diagnostic laboratory methods for onychomycosis vary in sensitivity and specificity. Direct mycological analysis and culture have an accuracy of 50% to 70%, depending on several variables, such as the way the collection was performed and the method of sample preparation [28]. False negatives may occur due to absence of viable fungi in the sample, and false positives may occur due to contaminant growth [29]. Thus, each of the analysis methods has its limitation; ideally, the analysis should be repeated, with new samples collected, for patients with a strong clinical suspicion of onychomycosis and whose first result was negative. Even so, in the present study, the combination of the three analyses allowed a considerable rate of positivity or confirmation of onychomycosis.

Anatomopathological analysis demonstrates utility in suspected cases where traditional mycological examinations (direct examination and fungal culture) are negative, or to exclude the diagnosis of psoriasis [28, 30]. The sensitivity of this test was higher than those traditionally performed when compared individually, reaching 85% [31]. Nevertheless, in the present study, the pathological analysis was positive for onychomycosis in 12 patients, whereas culture was positive in 16, so there was more diagnosis of onychomycosis in the cultures, showing the higher sensitivity of this test in relation to the others. However, there were six patients whose onychomycosis could only be diagnosed by histopathological analysis, while direct examination and culture were negative.

Anamnesis and detailed dermatological physical examination, careful collection of nail material for analysis, which included nail clippings and not only nail scraping, and the association of direct mycological analyses, culture, and pathology were factors that contributed to the results. Other important factors that may be cited are training and experience of technicians in collecting, processing, and analysis of the samples and interpretation of the results.

The study had some limitations, such as the sample number, which was relatively small to allow broader conclusions. In addition, it is noteworthy that the clinically suspected cases with negative results did not have repeated laboratory examination, which may have led to the loss of some cases [1, 32]. In this case, patient follow-up in longitudinal studies could lead to future diagnosis. Another point to consider is that only patients who were at least 30 days without antifungal drugs were included, as recommended by some studies [33, 34], However, other current studies have indicated suspension for 3–6 months due to the possibility of retention of the medication in subungual debris and its transfer to the culture medium, consequently inhibiting fungal growth [1, 35].

Our study reinforces the hypothesis that changes in psoriatic nails may facilitate local fungal infection. The use of immunosuppressive therapy may contribute to the increase in cases of onychomycosis, even more so with the recently available new immunobiological drugs. Overall, there are no robust studies available in the literature that have evaluated the role of immunosuppressive therapy as possible predisposing or contributing factors for fungal infections. Thus, further investigations are needed, including longitudinal and randomised studies with patients using or not using immunosuppressive drugs, to obtain further evidence on the relationship between onychomycosis and psoriatic disease.

## Figures and Tables

**Table 1:** Characteristics of the psoriatic patients.

Data	Patients (*n* = 38)	*p* value
*Gender*		0.7
Male	18 (47.4)	
Female	20 (52.6)	

*Psoriatic disease*		0.034
Psoriasis	15 (39.5)	
Psoriatic arthritis	4 (10.5)	
Both	19 (50)	

*Duration of clinical manifestations*		0.85
<1 year	0	
1–4 years	4 (10.5)	
5–9 years	8 (21.1)	
10–29 years	22 (57.9)	
>30 years	4 (10.5)	

*Duration nail changes*		0.92
<1 year	1 (2.6)	
1–4 years	10 (26.3)	
5–9 years	8 (21.1)	
10–29 years	15 (39.5)	
>30 years	2 (5.3)	
Do not know	2 (5.3)	

*Comorbidities*		0.16
None	13 (34.2)	
Systemic arterial hypertension	6 (15.8)	
Hypothyroidism	1 (2.6)	
Psychiatric disorder	2 (5.3)	
Benign nodule in the lungs	1 (2.6)	
SAH + DM	3 (7.9)	
SAH + dyslipidaemia	1 (2.6)	
SAH + CVI	1 (2.6)	
DM + HS	1 (2.6)	
Psychiatric disorder + Parkinson	1 (2.6)	
SAH + DM + dyslipidaemia	3 (7.9)	
SAH + DM + HS	1 (2.6)	
SAH + DM + hypothyroidism	1 (2.6)	
DM + GERD + megacolon	1 (2.6)	
SAH + DM + dyslipidaemia + hypothyroidism	1 (2.6)	
SAH + DM + dyslipidaemia + CVI	1 (2.6)	

*Nail manifestation * ^*∗∗*^		0.43
Subungual hyperkeratosis	13 (34.2)	
Onycholysis	11 (28.9)	
Hyperkeratosis + onycholysis	11 (28.9)	
Onychodystrophy	1 (2.6)	
Hyperkeratosis + onychodystrophy	1 (2.6)	
Onycholysis + onychodystrophy	1 (2.6)	

*Site*		0.88
Foot	21 (55.3)	
Hand	6 (15.8)	
Foot + hand	11 (28.9)	

SAH: systemic arterial hypertension; DM: diabetes mellitus*;* CVI: chronic venous insufficiency; GERD: gastroesophageal reflux disease; HS: hepatic steatosis. ^*∗∗*^Isolated or associated manifestations.

**Table 2:** Frequency of onychomycosis and laboratory results of nail analysis of psoriatic patients.

Data	Number	Frequency (%)
Onychomycosis confirmed	22	57.9
Positive direct mycological	17	44.8
Positive anatomopathological	12	31.6
Positive culture	16	42.1

**Table 3:** Fungi isolated in nail culture of individuals with psoriatic disease.

Fungus	Number of cases	Frequency (%)
Dermatophytes	9	56.25
*Trichophyton rubrum*	8	50
*Trichophyton tonsurans*	1	6.25

Yeasts	7	43.75
*Candida albicans*	3	18.75
*Candida parapsilosis*	4	25

**Table 4:** Drugs used to treat patients with psoriatic disease with and without onychomycosis.

Drugs	Number of patients, *n* (%)	With onychomycosis, *n* (%)	Without onychomycosis, *n* (%)
Acitretin	3 (7.89)	1 (2.63)	2 (5.26)
Methotrexate	14 (36.82)	13 (34.19)	1 (2.63)
Adalimumab	4 (10.52)	3 (7.89)	1 (2.63)
Etanercept	3 (7.89)	1 (2.63)	2 (5.26)
Infliximab	1 (2.63)	1 (2.63)	0
Secukinumab	2 (2.63)	0	2 (5.26)
Golimumab	2 (2.63)	0	2 (5.26)
Methotrexate + adalimumab	2 (2.63)	1 (2.63)	1 (2.63)
Methotrexate + etanercept	2 (2.63)	1 (2.63)	1 (2.63)
Methotrexate + infliximab	2 (2.63)	0	2 (5.26)
None	3 (7.89)	1 (2.63)	2 (5.26)
Total	38 (100)	22 (57.86)	16 (42.08)

## Data Availability

The research data used to support the findings of this study are available from the corresponding author upon request.
